# Rethinking empathy development in childhood and adolescence: a call for global, culturally adaptive strategies

**DOI:** 10.3389/fpsyg.2025.1575249

**Published:** 2025-08-26

**Authors:** Meshal A. Sultan, Nusrat N. Khan

**Affiliations:** ^1^Al Amal Psychiatric Hospital, Emirates Health Services, Dubai, United Arab Emirates; ^2^College of Medicine, Mohammed Bin Rashid University of Medicine and Health Sciences, Dubai Academic Health Corporation, Dubai, United Arab Emirates

**Keywords:** empathy, development, childhood, global mental health, culture, social–emotional learning

## Abstract

Empathy – the ability to recognize, understand, and respond to others’ emotions – is fundamental to human development and mental health. It unfolds across the lifespan, shaped by a complex interplay of biological maturation, social learning, and cultural context. Despite its universal importance, current clinical, educational, and policy frameworks often fail to integrate empathy-building interventions, particularly in low- and middle-income countries (LMICs) where resource constraints and cultural barriers hinder progress. This paper argues for a paradigm shift toward scalable, culturally adaptive strategies to foster empathy in diverse settings. We review developmental trajectories of empathy from infancy through adolescence, highlighting critical periods and influences, and examine practical interventions including caregiver–infant programs, school-based social–emotional learning (SEL), and clinician empathy training. We also address cross-cultural variations, proposing a framework to embed empathy-driven initiatives within healthcare, education, and policy. By prioritizing culturally sensitive, evidence-based approaches, global mental health systems can enhance therapeutic relationships, strengthen prosocial development, and address empathy gaps at a structural level. This perspective underscores an urgent need for interdisciplinary collaboration to position empathy as a cornerstone of global mental health initiatives.

## 1 Introduction

Empathy is a cornerstone of human social interaction, enabling individuals to resonate with and respond to the emotions of others. It involves interconnected processes, including emotion recognition, affective sharing (emotional contagion), and cognitive perspective-taking (mentalizing) (Chakrabarti and Baron-Cohen, 2006; Decety and Jackson, 2004). From an evolutionary standpoint, empathy is thought to have conferred survival advantages by enhancing cooperation and social bonding (Preston and de Waal, 2002). Indeed, signs of rudimentary empathy emerge remarkably early in life – for example, infants as young as 7 months can evaluate others’ social actions and show preference for prosocial over antisocial agents in simple animations (Geraci et al., 2024). Such findings suggest an innate predisposition for empathy development (Davidov et al., 2013).

However, empathy’s expression and impact are also modulated by context, varying across cultural norms, socialization, and individual differences (Eichbaum et al., 2023). These variations have important implications for mental health practice. For instance, certain neurodevelopmental and psychiatric disorders involve empathy deficits that require sensitive clinical approaches (Klapwijk et al., 2016). Unfortunately, the global mental health community – especially in many low- and middle-income countries (LMICs) – often overlooks empathy’s role, due to factors like scarce resources, limited training, and cultural stigma around emotional expression (Fitri et al., 2023). Bridging this gap calls for culturally nuanced, empathy-focused strategies in mental health care.

Humans are born with fundamental empathic capacities that provide a foundation, but these nascent capacities do not develop automatically; instead, children require nurturing social interactions to fully cultivate empathy. For example, supportive activities such as emotional communication with caregivers, social role-play, storytelling, and shared reading of age-appropriate literature are crucial for empathy to flourish (Bandura, 1977; Wagers and Kiel, 2019). Consistent with this, social neuroscience models distinguish between *affective empathy* (automatic, emotion-sharing processes) and *cognitive empathy* (deliberate perspective-taking), suggesting multiple componential development paths (Decety and Jackson, 2004; Lamm et al., 2007). These capacities are subserved by partly distinct brain circuits: affective empathy has been linked to activation in the anterior cingulate cortex and anterior insula (regions involved in experiencing and recognizing emotion), whereas cognitive empathy relies on regions such as the temporo-parietal junction (TPJ) and medial prefrontal cortex, which are crucial for theory of mind and perspective-taking (Lamm et al., 2007). Moreover, social learning and cultural theories note that children learn to express empathy through guidance from caregivers and societal norms – for instance, adults modeling empathic concern and culturally specific practices of emotion communication (Bandura, 1977; Vygotsky, 1978).

In light of these perspectives, empathy development can be seen as multi-determined: biologically primed yet highly sensitive to environmental shaping. In this article, we adopt an integrative view grounded in these theories. We propose that boosting empathy on a broad scale requires interventions targeting various levels – from individual caregivers to educational systems and policies – in ways that respect cultural contexts. We aim to enrich the discourse by examining empathy’s developmental trajectory, cultural variations, and implications for practice and policy, thereby clarifying how a global, culturally adaptive approach to fostering empathy can be realized.

This work is presented as a Perspective article – a conceptual analysis of empathy development from early childhood through adolescence – with an emphasis on culturally adaptive strategies to foster empathy in diverse contexts. We integrate developmental and cross-cultural insights to frame empathy as a global mental health priority.

## 2 Developmental aspects of empathy

Empathy develops through identifiable stages from infancy into adolescence, with each stage building on earlier capacities. Below we discuss key developmental phases – early infancy, childhood, and adolescence – noting how empathic abilities expand and what factors influence them.

### 2.1 Infancy and early childhood: precursors to empathy

Empathic behavior has its roots in infancy. Newborns and young infants exhibit emotional contagion, such as reflexively crying in response to other babies’ cries, indicating a primitive shared affect (Geangu et al., 2010). By a few months of age, infants engage in facial mimicry (copying caregivers’ emotional expressions) and show a clear preference for social stimuli (e.g., looking longer at faces than objects) (Farroni et al., 2002). These early behaviors are viewed as critical precursors to empathy, as they reflect an emerging ability to “resonate” with others emotionally.

Emotional synchrony between infants and caregivers – for example, a caregiver mirroring and appropriately responding to an infant’s signals – helps infants learn about emotions and builds the foundation for understanding others’ feelings (Levy et al., 2019). Notably, even at this young age, individual differences can be observed. For instance, research suggests that female infants, on average, display greater social interest (longer eye contact, more attentiveness to emotional cues) than male infants (Noonan et al., 2021). This heightened early social responsiveness in females may facilitate the accelerated development of empathy and other prosocial behaviors later in childhood. Conversely, infants who show atypically low social responsiveness may be at risk for difficulties: for example, infants later diagnosed with autism often exhibit reduced attention to others’ emotions in the first year of life, underscoring how important these early emerging capacities are (Hutman et al., 2010).

By the end of the first year, infants move beyond passive resonance and begin to show rudimentary empathic responses. Infants as young as 7–10 months demonstrate preferences for prosocial agents over aggressive ones, as shown in studies using simplified visual cues (Geraci et al., 2024; Kanakogi et al., 2013). While their responses likely rely on perceptual features rather than moral reasoning, such findings challenge the assumption that empathy only emerges after self-awareness. One possible mechanism is embodied simulation: through early imitation and the developing mirror neuron system, infants may vicariously experience others’ emotions (Gallese and Goldman, 1998). These early responses are supported by emerging joint attention between 9 and 12 months—such as following gaze or pointing—marking the infant’s growing ability to coordinate attention and share emotional experiences, which lays the groundwork for empathic understanding (Mundy and Newell, 2007).

This aligns with the views of other researchers that empathic concern does not depend on advanced cognitive self–other differentiation and is evident in the first year of life (Davidov et al., 2013; Kanakogi et al., 2013). Entering the toddler stage (around 1–2 years), children’s empathic repertoire expands further (Warneken and Tomasello, 2007). They begin to engage in prosocial actions such as comforting or helping others spontaneously (Warneken and Tomasello, 2007). Studies have documented that one-year-olds will attempt to soothe a crying peer or adult and will help caregivers in simple tasks like fetching out-of-reach objects (Warneken and Tomasello, 2007; Zahn-Waxler et al., 1992). These behaviors indicate that by the second year of life, most children not only feel with others but also act on that feeling to alleviate others’ discomfort.

Such early helping is initially quite context-bound – toddlers are more likely to help when adults explicitly communicate their need – but becomes more generalized with age (Svetlova et al., 2010). Overall, infancy and toddlerhood provide the building blocks of empathy: affective attunement, social interest, and the first instances of empathic concern and helping (Davidov et al., 2013). Interventions that promote caregiver–infant emotional attunement (e.g., sensitive response training for parents) can thus support empathy development from its very outset. This is particularly relevant in LMICs where formal resources are limited; empowering caregivers through community programs to engage in warm, responsive interactions can strengthen infants’ empathic foundations (Sultan, 2025).

### 2.2 Childhood: prosocial behaviors and cognitive empathy

As children grow through early and middle childhood, empathy becomes increasingly complex, transitioning from predominantly affective responses to including cognitive empathy. During the preschool years (~3–5 years old), children significantly improve in understanding that others have feelings, desires, and perspectives independent of their own. Although most children can grasp that someone else can hold beliefs that differ the child’s own beliefs by around 4 years of age (Wellman et al., 2001; Wimmer and Perner, 1983), recent studies show that precursors of Theory of Mind (ToM) are present much earlier. Infants as young as 18–26 months implicitly predict others’ actions based on what those others have or have not seen, indicating an early understanding of others’ perspectives (Barone et al., 2019; Schuwerk et al., 2021; Steffan et al., 2024). This emerging ability likely underpins early empathic responses. This new ability to infer others’ thoughts markedly enhances empathic capacity – children can now not only “feel with” others but also *imagine* others’ internal states. They start to succeed in perspective-taking tasks and can anticipate how their actions or words might affect someone else emotionally. Empirical studies link early ToM with prosocial behavior – likely because children better appreciate others’ viewpoints and needs (Imuta et al., 2016). These behaviors are supported by improvements in emotion regulation; as children learn to manage their own feelings, they can stay calm enough to focus on helping others (Eisenberg et al., 2010).

There is also a growing understanding of moral rules (e.g., fairness, not causing harm) that guides children’s empathic responses. For example, young children begin to say it’s “not nice” to hit someone because it hurts their feelings, reflecting integration of empathy into their moral reasoning (Ball et al., 2017). Parents and teachers play a critical role in this phase: studies show that children whose caregivers discuss emotions and encourage perspective-taking tend to develop stronger empathy and prosocial skills (Wagers and Kiel, 2019). Conversely, exposure to harsh or neglectful environments can blunt the growth of empathy, as seen in research on adverse childhood experiences impacting emotional development (Cerqueira and Almeida, 2023).

Scientific literature also indicates consistent gender differences in empathy through childhood, though these differences are modest compared to the overall growth all children experience. Girls, on average, score higher on measures of empathic concern and are often better at reading emotional cues, as reported in observational studies (Christov-Moore et al., 2014; McDonald and Kanske, 2023). They tend to outperform boys on tasks requiring emotion recognition or empathic responding. This may be partly due to socialization (girls may receive more encouragement to attend to others’ emotions) and partly due to developmental differences that appear early (as noted, infant girls show slightly greater social attunement) (Fivush et al., 2000). Boys are certainly capable of empathy, but some studies suggest they may express positive emotions and internalizing emotions less readily, possibly regulating their empathic reactions in line with cultural expectations of masculinity (Chaplin and Aldao, 2013). Importantly, individual variation is large, and many contextual factors (family climate, media, etc.) influence each child’s empathic development more than sex alone.

In summary, the childhood stage is when cognitive empathy truly blossoms. Children move from simple emotion contagion to understanding others’ perspectives and emotions in a nuanced way. Their empathic concern becomes more aligned with prosocial action – not only do they feel bad when others are sad, they increasingly try to help or comfort them. This solidifies empathy as a driver of positive social behaviors like sharing, cooperating, and moral reasoning. Educational programs in early childhood that integrate emotion training and perspective-taking (for example, classroom activities about recognizing feelings) have been shown to boost empathy and should be culturally adapted and implemented broadly (Aslan and Akyol, 2019).

### 2.3 Adolescence: integrating affective and cognitive empathy in social contexts

Adolescence is a pivotal period for empathy, marked by significant biological, cognitive, and social changes that together impact empathic abilities. During puberty and the teen years, brain regions involved in social cognition and emotion (such as the prefrontal cortex and limbic system) undergo remodeling, which can enhance certain aspects of empathy while also creating vulnerabilities (Konrad et al., 2013). Adolescents typically develop a more nuanced understanding of others’ emotions than children do, and their improving abstract thinking skills enable them to empathize with more complex situations (e.g., understanding the plight of people in different parts of the world or in hypothetical scenarios). Research indicates that overall empathy tends to increase from late childhood into adolescence, as youth become more socially aware and emotionally mature (Konrad et al., 2013).

However, the adolescent journey of empathy is not linear and can differ by gender and context. Gender differences often become more pronounced in adolescence. Adolescent girls, on average, report and display higher empathy—both affective empathy, like empathic concern, and smaller advantage in cognitive empathy, like perspective-taking—than boys (Christov-Moore et al., 2014; Mestre et al., 2009). This variance is thought to result from a combination of biological factors (e.g., different hormonal influences on emotion processing) and socialization processes (e.g., stronger encouragement for prosocial, communal behavior in females, whereas males may face peer norms that downplay emotional expressiveness) (Christov-Moore et al., 2014). These differences underscore the need for gender-sensitive approaches in fostering empathy: for instance, providing adolescent boys with structured opportunities (such as role-playing exercises or community service learning) can help practice and reinforce empathic skills in ways that are socially comfortable for them.

Another prominent aspect of modern adolescence is the influence of digital social contexts. Today’s adolescents develop empathy both face-to-face and through online interactions. Emerging research highlights a nuanced, bidirectional relationship between social media use and empathy. Moderate and active engagement on social platforms, such as sharing personal stories or offering support to peers, has been linked to increases in both cognitive and affective empathy over time (Vossen and Valkenburg, 2016). These online interactions may offer adolescents additional opportunities to practice perspective-taking and emotional responsiveness in diverse social contexts. However, it is equally important to recognize that adolescents with higher baseline empathy may be more inclined to engage in prosocial digital behaviors, suggesting a reciprocal relationship (Fu et al., 2022). At the same time, excessive reliance on digital communication may impair empathic development. Online exchanges often lack key non-verbal emotional cues, such as facial expressions or vocal tone, which are crucial for empathic accuracy. Experimental research has shown that limiting screen media significantly improved preteens’ ability to recognize non-verbal emotional cues (Uhls et al., 2014). Broader trends also raise concern: self-reported empathy levels among college students have declined in recent decades, a shift some researchers attribute partly to the rise of personal technology and reduced in-person social engagement (Konrath et al., 2011).

Over time, heavy users of social media may become less attuned to others’ non-verbal emotions, from lack of practice, or may experience “empathy fatigue” from constant exposure to others’ hardships on news feeds without the ability to tangibly help. Some evidence even suggests that the current generation of adolescents have self-reported lower empathic concern than prior generations, and researchers have speculated that increased digital media immersion could be a contributing factor. Some studies suggest a generational decline in empathic concern, possibly linked to digital immersion (Konrath et al., 2011). Still, longitudinal research by Vossen and Valkenburg (2016) found that social media use was associated with increased empathy when it supplemented real-life interaction (Vossen and Valkenburg, 2016). The effect of technology on empathy thus seems to depend on how and how much it is used.

Empathy develops qualitatively across childhood and adolescence, reflecting cognitive, social, and emotional growth. In early childhood, empathic responses tend to be concrete, immediate, and closely scaffolded by caregivers and cultural norms. Young children demonstrate concern for others, but their perspective-taking is often limited and egocentric. As children mature, cognitive empathy expands, particularly as executive functions and ToM abilities develop, allowing for a more flexible understanding of others’ emotions and beliefs (Decety, 2010; Van der Graaff et al., 2014). By adolescence, empathy becomes more abstract and socially nuanced, shaped by peer dynamics, media exposure, and emerging identity. Adolescents can hold multiple, even conflicting, perspectives and express concern for distant or hypothetical others. These shifts are underpinned by advances in cognitive control, emotional regulation, and moral reasoning, alongside increased exposure to diverse social experiences (Decety, 2010). Notably, longitudinal research highlights gender differences in developmental trajectories: girls typically show a steady increase in empathic concern, whereas boys may experience a temporary decline during early adolescence followed by a later rebound (Van der Graaff et al., 2014). These processes are summarized in Table 1, which outlines the developmental trajectory of empathy in tandem with contextual and neurocognitive changes.

**Table 1:** Developmental differences in empathy across infancy, childhood, and adolescence.

Developmental stage	Social context and cultural factors	Empathic characteristics	Key cognitive/social functions supporting empathy
Infancy (0–2 yrs)	Primary context is family; empathy socialization via caregiver-infant interactions. Cultural practices (e.g., caregiver closeness, communal caregiving) influence emotional attunement.	Affective: Rudimentary empathy (emotional contagion, reacting to others’ distress by reflex). Cognitive: Precursor skills in early infancy (self-other not fully differentiated). By the end of the first 2 years children show implicit awareness of others’ beliefs/perspectives (precursors of Theory of Mind).	Joint attention emerges (6–12 mos) – infant and caregiver share focus, building social understanding. Emotion regulation: minimal (infant relies on caregiver to soothe). Prosody/pragmatics: infant tunes into emotional tone of voices.
Early Childhood (3–5 yrs)	Family and preschool are key contexts; children begin learning cultural norms about caring (e.g., sharing, “be nice”). Adults (parents, teachers) model and reinforce empathy.	Affective: Displays of empathic concern (may comfort or help others spontaneously, though empathy is often egocentric, e.g., offers their own doll when someone is sad). Cognitive: Further development of Theory of Mind (~4 yrs. old) – child realizes others have feelings and thoughts independent of their own. Empathy mostly for familiar people; difficulty with multiple perspectives.	Emotion vocabulary: expands (learns names for feelings, improving communication of empathy). Inhibitory control: improving but still developing (can sometimes inhibit impulses to avoid hurting others, but not always). Mental flexibility: limited; thinking is still centered on one perspective at a time. Pragmatics: learns social rules like saying “sorry” or giving hugs when someone is upset.
Middle Childhood (6–11 yrs)	Peers and school life become more influential alongside family. Empathy is encouraged by group activities, teamwork, and cultural values (e.g., responsibility for younger siblings in some societies). Sociodemographic factors (such as having to care for siblings or exposure to diversity at school) can broaden empathic understanding.	Affective: More consistent empathy; can feel concern for others in wider contexts (e.g., classmates, fictional characters). Can experience empathy even when not personally responsible (sympathy for someone who fell on the playground, even if the child wasn’t involved). Cognitive: Perspective-taking grows; can understand more complex emotions (e.g., that someone can be sad and angry at the same time). Starts to grasp others’ viewpoints in conflicts. Empathy begins extending to social groups (fairness, justice notions start, e.g., “It’s not fair if someone is left out”).	Perspective-taking: solidifies (can take another’s perspective in concrete situations). Joint attention: well-established, now used in group settings (e.g., team sports, group projects). Inhibitory control: much improved (better at controlling own anger or excitement to respond empathically). Emotional literacy: understands nuanced emotions (pride, guilt) which aids empathic accuracy. Moral reasoning: shifts from focus on punishment to understanding impact on others (e.g., “If I hurt him, he’ll feel bad,” indicating empathic moral reasoning). Mental flexibility: improving (can handle multiple pieces of social information).
Adolescence (12–18 yrs)	Peer group, societal and cultural identity are highly significant. Adolescents are exposed to broader social issues (via media, education). Culture and subculture (e.g., youth culture, community values) shape where empathy is directed (family vs. friends vs. global causes). In LMICs, adolescents may take on adult roles early, influencing empathic responsibilities.	Affective: Capable of empathizing with distant others or abstract groups (e.g., feeling empathy for people affected by a natural disaster in another country). Greater ability to manage personal distress and channel empathy into supportive action (e.g., comforting a friend, activism). However, emotional empathy might be moderated by peer norms (some teens hide empathetic feelings if their peer culture discourages outward emotion). Cognitive: Advanced perspective-taking (understands complex, even opposing perspectives; can do meta-perspective, “I understand that he sees it differently because of his background”). Theory of Mind is fully developed, including second-order beliefs (“I think that she thinks…”). Empathy is more principled – often tied to adolescents’ developing moral and philosophical views.	Theory of Mind: mature (adolescents understand ambiguity, sarcasm, others’ inner conflicts). Perspective-taking: refined (can empathize with people from different cultures or life experiences, given knowledge). Mental flexibility: high (can integrate multiple social cues and perspectives). Inhibitory control: near adult levels (can suppress inappropriate reactions; e.g., stays calm to support a friend in crisis). Pragmatics of empathy: knows how to express empathy suitably (e.g., when a hug is appropriate vs. when words are better). Moral reasoning: often at “social contract” or “universal ethics” stage – empathy underpins values of justice and care. Prosody: Adolescents can detect subtle tone differences and implied emotions, aiding empathic understanding in communication.

In conclusion, adolescence is a time of refining empathy: teens integrate the emotional empathy of childhood with the new cognitive and abstract capacities of their maturing minds, all while navigating complex social dynamics. Interventions for adolescents can leverage their expanding cognitive abilities and desire for social connection. For instance, programs that involve peer discussions of moral dilemmas, perspective-taking activities, or service projects can channel adolescent idealism and identity exploration into empathic growth. It is also crucial for parents, educators, and clinicians to remain attuned to how adolescents engage digitally, guiding them toward healthy online interactions that reinforce (rather than replace) real-world empathy.

### 2.4 Applied strategies to support empathy development

Across all stages of development, empathy can be nurtured through age-appropriate strategies in family, educational, and clinical settings. In infancy, caregiver–infant engagement is key. Responsive parenting—mirroring emotions, naming feelings (“You’re smiling, you must be happy!”), and offering comfort—helps infants develop emotional synchrony and trust. Simple face-to-face play and joint attention activities (e.g., pointing out a puppy and saying “Look, we see it together!”) support the infant’s capacity to share experiences, laying the foundation for empathy (Mundy and Newell, 2007).

In early childhood, families and schools both play key roles. Parents can foster empathy by discussing emotions (“How do you think your friend felt when you took the toy?”), reading storybooks with reflection on characters’ perspectives, and modeling kindness. Educators can build on these skills with cooperative classroom activities and social–emotional learning (SEL) programs that teach emotional recognition, turn-taking, and comfort-giving. Structured lessons on perspective-taking have been shown to significantly enhance empathic skills in young children (Aslan and Akyol, 2019). Clinicians may also support development through guided role-play or play therapy, especially for children struggling with empathy.

In adolescence, face-to-face social interaction remains central to empathy development, as it provides opportunities for nuanced emotional engagement and perspective-taking. According to Hoffman’s developmental theory, adolescence marks a transition toward empathy for others’ life conditions, in which individuals can engage with abstract social issues and imagine the experiences of distant others (Hoffman, 1979). Structured, in-person activities, such as volunteering or mentoring, can cultivate these advanced empathic capacities by exposing adolescents to diverse perspectives. Longitudinal research by Van der Graaff et al. (2014) shows that empathic concern and perspective-taking generally increase throughout adolescence, particularly when youth are embedded in supportive social environments. Complementary digital tools may have value but should not displace embodied, real-world experiences that serve as the foundation for empathic learning.

By tailoring empathy-building efforts across developmental stages, from responsive caregiving in infancy to structured social engagement in adolescence, empathy can be cultivated progressively. Each phase builds upon the last, guiding children toward a mature, well-rounded empathic capacity.

## 3 Cross-cultural perspectives on empathy

Empathy is a universal capacity expressed through culturally specific practices. Cultural norms shape how empathy is socialized, interpreted, and displayed across development. In Western individualistic cultures, empathy is often cultivated through explicit emotional expression and perspective-taking—for example, encouraging children to “put themselves in someone else’s shoes” (Eichbaum et al., 2023). In contrast, collectivist cultures such as many in Asia, Africa, and the Middle East tend to promote empathy through relational harmony, non-verbal sensitivity, and fulfilling social roles. In these contexts, empathy may emerge implicitly, for example by teaching respect for elders and attentiveness to others’ needs rather than open emotional disclosure (Markus and Kitayama, 1991). Neither cultural style implies greater or lesser empathic ability, but the outward expressions and mechanisms of empathy differ meaningfully by context.

Such cultural variations have important implications for mental health and education. One-size-fits-all approaches to empathy promotion often fail when applied cross-culturally. Programs that encourage open emotional discussion may resonate in some contexts but require adaptation elsewhere—for example, using stories, proverbs, or group rituals to convey empathic values in cultures where emotional restraint is normative (Hodge et al., 2002). School-based SEL programs in LMICs have shown promise when locally adapted (Lee, 2018).

Empathy-building strategies are most effective when adapted to the sociocultural context in which they are delivered. For example, in collectivist societies or LMICs where extended families are common, children often acquire perspective-taking skills through everyday interactions with siblings, cousins, and community members (Hoffman, 1979). These organic learning environments may provide informal but repeated exposure to diverse viewpoints, promoting the development of cognitive empathy. In contrast, more individualistic contexts may benefit from structured approaches, such as reflective peer groups modeled after Balint groups. Such interventions, when adapted for use by community health workers or educators, can facilitate empathetic dialog grounded in shared cultural experiences. Decety (2010) emphasizes that adolescence brings neurodevelopmental changes, particularly in the prefrontal cortex, that support abstract reasoning and social cognition, making this a sensitive period for targeted empathy training. Moreover, tools like the Empathy Questionnaire (EmQue) developed by Rieffe et al. (2010) highlight that even young children express emotional contagion and prosocial concern in culturally variable ways, suggesting that intervention formats should reflect local socialization practices. For instance, a role-play activity in one culture might require group-based adaptation in another, or joint attention might emerge through different caregiving routines. Aligning empathy-building efforts with lived experiences ensures greater resonance and sustainability.

## 4 Implications

Understanding empathy’s development and cultural context has important implications for practice and policy in mental health and education. We outline recommendations in three domains: clinical practice, education/training, and policy. In all areas, we emphasize evidence-based approaches that are scalable and culturally adaptive, in line with the need to globalize empathy-building efforts.

### 4.1 Clinical practice

Understanding empathy’s development supports targeted interventions across age groups. In early childhood, atypically low empathic concern may indicate risk for neurodevelopmental or behavioral disorders (Fatima and Babu, 2024), although most such associations remain correlational rather than causal. Routine assessments, such as asking whether the child attempts to comfort others, can help flag concerns, especially when supported by validated screening tools. For example, the EmQue assesses emotional contagion, attention to others, and prosocial actions in young children, offering a structured way to observe early empathic tendencies (Rieffe et al., 2010). In typically developing children, responsive caregiving and emotion coaching are associated with increased empathy and fewer behavior problems, thereby supporting socio-emotional growth (Liu et al., 2022). In LMICs, scalable community-based training, such as the World Health Organization (WHO) Caregiver Skills Training Program and the WHO Health Promoting Schools Framework, have been implemented to enhance caregiver–child interaction and promote social engagement in children (Salomone et al., 2019; Harte and Barry, 2024). These interventions empower caregivers with practical, responsive strategies that foster shared attention and emotional reciprocity (Salomone et al., 2019; Harte and Barry, 2024).

For adolescents, empathy-based interventions can reduce aggression and promote prosocial behavior (Castillo et al., 2013). Programs like Roots of Empathy, in which an infant is introduced into the classroom to stimulate perspective-taking, have shown improvements in students’ prosocial behavior and modest increases in self-reported empathy across diverse cultural contexts (Connolly et al., 2018). Cognitive-behavioral therapy that integrates structured perspective-taking exercises can enhance social understanding, especially in youth with conduct challenges (Matthys and Schutter, 2021). Volunteering and youth service programs have also been linked to increased empathic concern and reduced in-group bias. For instance, young adults who participated in community volunteering scored significantly higher on dispositional empathy compared to non-volunteers (Nowakowska, 2022).

In clinical practice, clinicians’ own empathy significantly impacts outcomes. Strong therapeutic alliances, founded on empathic listening and attunement, are correlated with increased treatment adherence and patient satisfaction (Liber et al., 2010). Cultural empathy, which involves recognizing and responding to patients’ unique sociocultural backgrounds and communication styles, has been shown to enhance rapport and facilitate more effective care delivery in multicultural contexts (Ullrich, 2019).

Clinicians should also attend to adolescents’ digital environments. Social media can shape empathy development in both positive and negative ways. While excessive online engagement may limit emotional cue recognition, supportive digital interactions, such as expressing care or sharing stories, may foster perspective-taking. Encouraging discussions about online behavior and guiding youth toward meaningful, prosocial engagement can help strengthen empathy in the digital space (Vossen and Valkenburg, 2016).

### 4.2 Education and training

Empathy education should begin early and span child, adolescent, and professional development. SEL programs integrated into curricula are associated with gains in emotional literacy, empathy, academic performance, and social behavior (Van Pham, 2024). Meta-analyses indicate durable, cross-cultural benefits across grade levels (Cipriano et al., 2023; Corcoran et al., 2018). Culturally responsive adaptation, through co-design with community stakeholders, improves fit and effectiveness, particularly in under-resourced urban settings (Kurtz et al., 2023) and in low-income or crisis-affected contexts where implementation often hinges on cultural relevance, dosage, and policy support (McCoy and Hanno, 2023).

Healthcare professionals also benefit from structured empathy training that builds perspective-taking and emotional insight. Role-play of clinician–patient encounters and guided feedback develop empathic communication through experiential learning, while narrative approaches (e.g., reflecting on patient stories) deepen emotional attunement and have shown improvements in empathic responsiveness, especially among nurses (Adamson et al., 2018). Balint groups, facilitated discussions of emotionally complex cases, offer a collaborative forum to explore provider and patient perspectives; a recent meta-analysis of randomized trials found significantly greater increases in clinicians’ self-reported empathy among participants compared with controls (Gong et al., 2024). These activities reinforce empathy as a professional skill, strengthen the therapeutic alliance, and align with broader training innovations (Adamson et al., 2018; Banks, 2025).

Adapting empathy-building to digital contexts is especially important in LMICs, where traditional resources may be limited but mobile access is widespread. Digital storytelling can make socio-emotional content concrete and engaging; experimental evidence with young children shows greater empathy gains from interactive digital stories than from traditional storytelling (Maranatha et al., 2024). To ensure feasibility and equity, facilitators in LMICs can blend online and offline components (e.g., WhatsApp reflections following in-person activities), while attending to implementation barriers such as cultural fit, time for practice, and institutional support (McCoy and Hanno, 2023). Where national policy enables routine classroom time for empathy (e.g., dedicated weekly sessions), integration and sustainability are further facilitated (Stoltzfus, 2016).

### 4.3 Policy recommendations

Empathy should be embedded in national education strategies and public health planning. For example, Bhutan’s “Gross National Happiness” framework incorporates compassion and emotional development into mainstream education (Drukpa, 2016). Similarly, Denmark has made empathy training a formal part of its national curriculum, dedicating a weekly hour (“Klassen Time”) for school students to practice social problem-solving and perspective-taking (Stoltzfus, 2016). These policies illustrate how national frameworks can prioritize SEL to promote social trust and well-being.

Public health policy can also support parenting interventions that foster empathy in early childhood. Programs such as “Reach Up and Learn” provide play-based coaching to caregivers to promote early development (Wilton et al., 2023), and WHO’s Caregiver Skills Training program teaches responsive strategies to support children’s social engagement and emotional understanding (Wong et al., 2022). However, implementing such programs in low-resource settings presents practical challenges. In many low- and middle-income countries (LMICs), educational and healthcare systems prioritize urgent needs like literacy, vaccination, and nutrition. Consequently, SEL and empathy-related initiatives may be seen as peripheral. For instance, while Reach Up and Learn shows promise in promoting developmental outcomes, scaling such programs nationally is often constrained by limited budgets and workforce shortages.

To improve feasibility, empathy-building strategies can be integrated into existing services. Training teachers or community health workers to deliver brief, culturally relevant empathy activities during routine school lessons or health visits can reduce implementation barriers while enhancing outcomes. As McCoy and Hanno note, successful SEL policy implementation requires cultural alignment, consistent support, and integration into broader systems (McCoy and Hanno, 2023). Political will and institutional backing are essential to sustain such efforts over time.

Community-led initiatives, such as storytelling circles, youth service clubs, or digital empathy projects, also hold promise for informal education (Fiddian-Green et al., 2023; Maranatha et al., 2024). Moreover, cross-cultural research is urgently needed. The existing evidence base on empathy development and training remains heavily Western-centric (Takamatsu et al., 2021). Expanding studies to include underrepresented contexts will help identify culturally specific protective and risk factors and strengthen the global relevance of policy recommendations. Notably, Chopik et al. (2017) documented cultural variations in empathic concern across 63 countries, underlining the need to contextualize empathy initiatives. High-level policy documents should thus recognize empathy not as an optional add-on but as foundational to social cohesion, civic responsibility, and collective resilience.

## 5 Discussion and conclusion

This perspective has explored empathy’s development from infancy through adolescence, highlighting its early biological roots and the profound influence of social and cultural context (Tousignant et al., 2017). While infants show proto-empathic behaviors, empathy matures through experience and interaction. Across development—from toddlers’ helping behaviors to adolescents’ digital social lives—empathy emerges as a dynamic, context-sensitive capacity.

Empathy is not only a personal capacity but a critical factor in the quality of social relationships throughout development. In childhood, higher empathy is associated with more positive peer interactions—children who understand and share feelings are more likely to cooperate, help others, and form trusting friendships. In contrast, children with lower empathy may struggle socially and exhibit more aggressive or unresponsive behaviors. In adolescence, empathy continues to shape interpersonal dynamics: empathetic teens tend to engage in more prosocial behaviors, resolve conflicts through perspective-taking, and show stronger moral engagement, while those with low empathy may experience peer conflict, show callous attitudes, or engage in antisocial behavior. These patterns highlight the social value of fostering empathy early, not only to support individual development but also to strengthen community well-being by reducing aggression and isolation.

Regarding the scope of this perspective, we focus specifically on childhood and adolescence. We do not address empathy development in adulthood. We do not attempt to include every neural mechanism or cultural nuance but attempt to provide a more general overview. Additionally, much of the evidence cited originates from high-income countries, so our global recommendations should be applied with cultural sensitivity. We emphasize the need for more cross-cultural research and locally grounded data from LMICs to validate and refine the strategies proposed.

To support practical application, we offer recommendations for educators, parents, and professionals. Educators can embed empathy-building into daily routines through SEL activities like classroom discussions, role-play, and perspective-taking projects. Parents can engage in emotion coaching at home—discussing feelings, modeling kindness, and using shared reading to explore characters’ emotions—all of which foster empathic concern. Mental health professionals can benefit from empathy training (detailed in Section 4.2) and should model empathy in their clinical practice. Community leaders and policymakers can reinforce these efforts by integrating empathy objectives into youth programs or national education standards. These concrete strategies can help translate conceptual insights into meaningful action across schools, homes, clinics, and communities.

The transition from childhood to adolescence is shaped by whether empathy has been nurtured early on. Adolescents who developed empathy in childhood tend to show greater perspective-taking, emotional attunement, and concern for others. They often consider the impact of their actions (“Will this hurt my friend?”), feel appropriate guilt or sympathy, and regulate aggression more effectively. Longitudinal studies link early empathic concern with increased prosocial behavior and moral reasoning in adolescence—empathic teens are more likely to help peers and stand up against bullying (Ball et al., 2017).

In contrast, adolescents who lacked early opportunities to develop empathy may focus primarily on their own needs, with little emotional response to others’ distress. They may struggle to form close relationships, appear indifferent or callous, and are at higher risk for antisocial behavior. Research on callous-unemotional traits shows that low empathy in childhood predicts persistent behavior problems into adolescence and beyond. These youth often fail to grasp why certain actions are wrong unless external consequences are involved, reflecting impaired moral development. A longitudinal study observed that children with low concern for others were more likely to show chronic conduct problems over time (Hastings et al., 2000).

In sum, early empathy development equips adolescents with essential cognitive and emotional tools for healthy relationships and ethical decision-making. Without it, teens are more likely to face social difficulties, emotional disconnection, and behavioral challenges.

A core implication is that empathy should be deliberately nurtured through policy and practice. Interventions can be effective across life stages: early childhood programs leverage neural plasticity, school-based SEL refines socio-emotional skills, and adolescent-focused efforts can redirect growing social awareness into prosocial behavior. Cultural adaptation is essential; programs must align with local norms to ensure relevance and sustainability (Sultan, 2025). Case examples from Malawi demonstrate that community-driven approaches can successfully foster empathy even in low-resource settings (Lee, 2024).

This work reflects ecological and social learning frameworks: children’s empathic growth depends on layered environments—families, schools, communities, and media. Integrating empathy into clinical and educational systems is not only evidence-informed but ethically grounded in humanistic psychology, recognizing empathy as a cornerstone of well-being. Empathy-focused systems may also help reduce violence, discrimination, and alienation.

We call for a paradigm shift—particularly in under-resourced regions where empathy has been overlooked—to prioritize empathy in research, healthcare, and education. Embedding empathy-building strategies within these systems, and tailoring them culturally, can promote more inclusive schools, better therapeutic relationships, and stronger communities.

Promoting empathy is not an abstract ideal; it is a practical response to global challenges requiring cooperation and mutual understanding. Advancing this agenda demands interdisciplinary collaboration—from psychologists to educators, clinicians to policymakers. By centering empathy as a developmental and societal priority, we can cultivate the capacities needed for a more connected, compassionate, and resilient world.

## Data Availability

The original contributions presented in the study are included in the article/supplementary material, further inquiries can be directed to the corresponding author.
